# Monthly Summary

**Published:** 1887-02

**Authors:** 


### Monthly Summary.

---

RESTORING A BICUSPID.—*By A. H. Hilzim.*—A left upper bicuspid, the bucal cusp was entirely gone, even up to the margin of the gum. Some one had endeavored to fill this tooth, and partially restored the contour with amalgam, which did not answer the description of the various "white alloys" now advertised, as it was quite *black* and unsightly. As it was "leaking" all around its edges, and very sensitive to "sweet things," I removed the filling.

The work of removal was done very carefully, as there was danger of breaking the remaining cusp, and this would have spoiled the operation I had in view. The great black lump of amalgam removed, I found, fortunately, the nerve not exposed.

You will say why not contour the tooth with gold? But the patient objected to so much gold showing, almost as much as the amalgam, besides I suggested a better plan. I first undercut the remaining cusp as much as possible, and prepared the tooth throughout, as if for filling. I took a piece of pattern metal about one-sixteenth of an inch wide, and long enough to encircle the tooth; fitted it around nicely, removed it, and patterned from it a strip of gold, from a piece kept for such purposes, and having bent it to conform, as nearly as possible to the shape of the tooth, soldered it smoothly together.

After beveling the edges, and polishing this band nicely, it is placed in position over the broken tooth, and with the automatic mallet driven home. When in position, the upper edge of the band is slightly under the margin of the gum, and the lower extends down over the broken surface of the tooth, and forms a socket or receptacle for—well anything you may choose to insert.

However, my plan at this juncture was to take a short cuspid (rubber tooth) and grind the neck of it, so as to fit nicely down within the band, and at the same time to articulate with the lower teeth, and to fall into line with, and conform in appearance to, the upper teeth.

Oxyphosphate is then mixed to a proper consistency, placed within the band, and the cuspid forced into this and held in position, till the phosphate sets firmly around it. Now, with gold, or a good article of amalgam, fill in round the pins of the artificial cusp, and in the undercut of the natural one, and you have something for your pains, which your patient and you will be well pleased with.—*Miss. Trans.*

---

REMEDY FOR TIRED EYES.—People speak about their eyes being tired, meaning that the retina, or seeing portion of the eye, is fatigued, but such is not the case, as the retina hardly ever gets tired. The fatigue is in the inner and outer muscles attached to the eye-ball and the muscle of accomodation, which surrounds the lens of the eye. When a near object is to be looked at this muscle relaxes, and allows the lens to thicken, increasing its refractive power. The inner and outer muscles are used in covering the eye on the object to be looked at, the inner one being especially used when a near object is looked at. It is in these muscles mentioned that the fatigue is felt, and relief is secured temporarily by closing the eyes or gazing at far-distant objects. The usual indications of strain is a redness of the rim of the eyelid, betokening a congested state of the inner surface, accompanied with some pain. Sometimes this weariness indicates the need of glasses rightly adapted to the person, and in other cases the true remedy is to massage the eye and its surroundings as far as may be with the hand wet in cold wator.—*Herald of Health.*

---

LOCAL REMEDY FOR NEURALGIA—A mixture of one part of iodoform to ten or fifteen of collodion, if spread repeatedly upon a neuralgic surface until it attains a thickness of one or two millimetres, is said to be quite effective in the treatment of certain neuralgias. If the first application does not speedily terminate the neuralgia, those who have used this mode of treatment direct that its application should be continued. It seems especially valuable in the relief of neuralgias of the trigiminus. It also seems of value to be applied along the spine, particularly at painful points in what is called spinal irritation. These observations are by no means new, and yet they seem worthy of further consideration.—*Neurological Review.*

---

A DISINFECTANT.—A new disinfecting compound for puri ying the atmosphere of the sick room has just been presented to the Berlin Medical Society. Oils of rosemary, lavender and thyme, in the proportion of 10, 2½, and 2½ parts respectively, are mixed with nitric acid in the proportion of 30 to 1½. The bottle should be shaken before using, and a sponge saturated with the compound and left to diffuse by evaporation. Simple as it is, the vapor of this compound is said to possess extraordinary properties in controlling the odors and effluvia of offensive and infectious disorders.—*Pacific Record of Med. and Phar.*

